# Supplementary material for: Infrared spectroscopy data- and physics-driven machine learning for characterizing surface microstructure of complex materials
Source: Nat Commun. 2020 Mar 23;11:1513. doi: 10.1038/s41467-020-15340-7 (PMC7089992; doi:10.1038/s41467-020-15340-7)
Supplement: Supplementary file 1 — Supplementary Information [file 41467_2020_15340_MOESM1_ESM.pdf]

Infrared Spectroscopy Data- and Physics-driven Machine Learning for Characterizing Surface Microstructure of Complex Materials

Supplementary Information

Lansford et al.

Table of Contents

Supplementary Note 1: First-Principles Data and Physical Models 2
Supplementary Note 2: Coverage Scaling Parameters and Extension to Pt-CO Frequencies .. 3
Supplementary Note 3: Quantifying Structure with PDFs 4
Binding-type pdf 4
Generalized coordination number (GCN) pdf 4
Supplementary Note 4: Overview of Data Variability 5
Supplementary Note 5: Learning Discrete PDFs with Multinomial Regression 6
The softmax output activation 6
The Wasserstein loss function 6
Supplementary Note 6: Choice of Algorithm and Deriving the Wasserstein Derivative 7
Choice of algorithm 7
Derivative of the Wasserstein squared with respect to the softmax 7
Supplementary Note 7: Hyperparameter Tuning and Model Assessment 9
Supplementary Note 8: Explanation of Experimental Spectra 14
Supplementary Note 9: Details of First-Principles Calculations 15
Supplementary Note 10: Extension with NO as a Probe Molecule 17
Supplementary Note 11: Step by Step Instructions for the Spectra to Structure Software .. 20
Using existing trained neural networks or neural network ensembles 21
Generating one's own primary dataset 21
Training one's own neural network and running cross validation trials 22
Supplementary References 23

Supplementary Note 1: First-Principles Data and Physical Models

We use spectra from well-characterized systems where the coverage is known precisely, along with the overlayer structure when relevant. Applying Equations 1 and 2 of the main text to the data in Supplementary Table 1 and Supplementary Table 2 results in scaling factors (SF) for the C-O stretch frequency and Pt-CO vibrations being 1.012 with an uncertainty of 0.002 and 0.969 with an uncertainty of 0.0035, respectively. These scaling factors are applied to all the frequencies and intensities calculated from DFT used in our model.

Supplementary Table 1 | Experimental and DFT peak positions for the C-O stretch frequency from the spectra corresponding to well-characterized coverages and ordered overlayers. Also shown is the binding-type of the corresponding peak, the DFT value after scaling by the C-O scaling factor, and the error in the frequency after applying the scaling factor.* indicates a DFT frequency adjusted to account for a difference in the coverage of the DFT calculation from the experiment. Any adjustments were computed using the coverage scaling relationship developed in this work.

Pt facet	Overlayer Structure	Absolute Coverage [ML]	Binding-Type	Experiment [cm^{-1}]	DFT [cm^{-1}]	Scaled DFT [cm^{-1}]	Error [cm^{-1}]
(111)	None	0.17	atop	2080[1]	2055*	2080	-0.47
(111)	c(4x2)	0.5	atop	2105[2]	2071	2096	-8.92
(111)	c(4x2)	0.5	bridge	1855[2]	1835	1857	2.22
(111)	c(4x2)	0.55	atop	2105[3]	2075*	2100	-5.07
(111)	c(4x2)	0.55	bridge	1854[3]	1844*	1866	11.87
(110)	p(2x1)	1.0	atop	2094[4]	2068	2093	-0.95
(100)	c(2x2)	0.5	atop	2087[5]	2064	2089	2.00
(100)	c(4x2)	0.75	atop	2091[6]	2068	2093	2.05
(100)	c(4x2)	0.75	bridge	1901[6]	1877	1900	-1.27

Supplementary Table 2 | Experimental and DFT peak positions for the Pt-CO stretch frequency from the spectra corresponding to well-characterized coverages and ordered overlayers. Also shown is the binding-type of the corresponding peak, the DFT value after scaling by the Pt-CO scaling factor, and the error in the frequency after applying the scaling factor.* indicates a DFT frequency adjusted to account for a difference in the coverage of the DFT calculation from the experimental value. Any adjustments were computed using the coverage scaling relationship developed in this work.

Pt facet	Overlayer Structure	Absolute Coverage [ML]	Binding-Type	Experiment [cm^{-1}]	DFT [cm^{-1}]	Scaled DFT [cm^{-1}]	Error [cm^{-1}]
(111)	None	0.17	atop	472[7]	489	474	2.10
(111)	c(4x2)	0.5	atop	464[8]	478	463	-1.05
(111)	c(4x2)	0.5	bridge	376[8]	386	374	-2.16
(110)	p(2x1)	1.0	atop	472[4]	488	473	0.63

Experimental frequencies used in computing the SFs correspond to the maximum intensity of the experimental spectrum in the range relevant to the target CO stretch. These experimental frequencies, often referred to as the peak locations, are on the order of 2000 and 400 cm^{-1} for the C-O and Pt-CO stretches, respectively. To obtain ω for the purposes of computing the frequency scaling factors, the DFT intensities are convoluted with a Gaussian function having a full width half maximum (FWHM) of 45 cm^{-1}.

In Supplementary Figure 1 we show that DFT can accurately generate relative intensities of IR spectra. Shown are the experimental spectra (green solid line) and experimental spectra[1] (blue dashed line). No coverage scaling factors were applied to this data as the CO c(4x2) overlayer on Pt(111) was modeled exactly via DFT using 8 CO molecules adsorbed on a slab consisting of 16 Pt atoms. Frequency scaling factors were not applied here because the aim is to show the ability to generate relative intensities. Application of frequency scaling factors would shift the DFT computed spectra to the right so that it more closely overlays the experimental spectra. Half of the CO molecules

are on atop sites and half on bridge sites. The computed spectra is accurate in both the high-frequency C-O modes and the lower frequency Pt-CO modes for both atop and bridge sites.

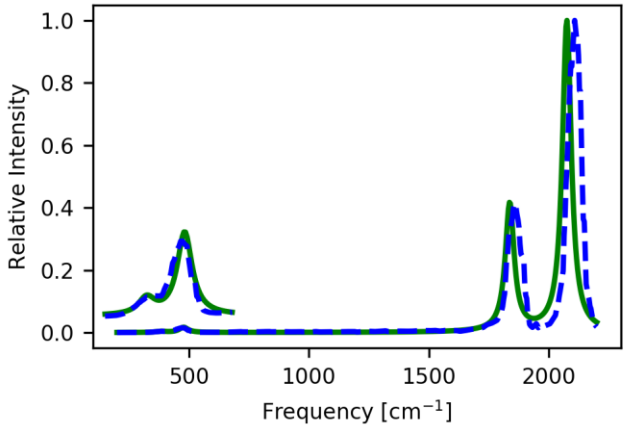

Supplementary Figure 1 | DFT-based calculated spectra (green line) for CO on Pt(111) at 0.5 ML in a c(4x2) configuration and experimental spectra (blue dashed lines). Plotted is the relative intensity vs. wavenumber. The DFT-calculated frequencies and intensities were convoluted with a Lorentzian with 45 cm^{-1} FWHM. The experimental spectra was taken from HREELS at UHV and scaled by frequency raised to the 2.7 to convert to IR spectra.

Supplementary Note 2: Coverage Scaling Parameters and Extension to Pt-CO Frequencies

Coefficients regressed on data in Figure 4 to Equation 3 of the main text using OLS on high coverage, extended-surface DFT data and associated R^2 values are shown in Supplementary Table 3. Experimental isotopic studies cannot be used to produce linear scaling of coverages as similar masses of available isotopic species result in coupling of their normal modes[9]. In the spirit of experimental isotopic studies, however, we can populate the surface computationally with isotopes of different masses to mimic the low-level of coupling between CO molecules adsorbed at different binding-types. In this way, we expand our high coverage dataset on extended surfaces from 43 to 173 data points and generate frequency-coverage scaling relations that depend linearly on site-specific (SS) spatial coverage and total (T) spatial coverage. To remove ambiguity in our definition of coverage, only ordered overlayers in which all adsorbates are equally spaced are used in OLS.

Supplementary Table 3 | Coverage scaling factor (CSF) regression coefficients for scaling C-O frequencies from zero to elevated coverage. Coefficients α_{ss} multiply spatial coverage of similar sites and coefficients α_T multiply total spatial coverage.

Binding-Type	α_{ss} [Å^2 per CO]	$\sigma(\alpha_{ss})$	α_T [Å^2 per CO]	$\sigma(\alpha_T)$	R^2
Atop	0.374	0.018	-0.094	0.011	0.937
Bridge	0.348	0.052	0.294	0.035	0.966
3-fold	0.449	0.055	0.390	0.039	0.989
4-fold	0.353	0.171	0.587	0.114	0.965

The coverage dependence of Pt-CO frequencies and intensities for CO adsorbed at atop sites are illustrated in Supplementary Figure 2a and b, respectively. The absolute value of intensity for Pt-CO normal modes at all binding-types is shown in Supplementary Figure 2c. Intensities at hollow sites are lower than at atop and bridge sites and more constant.

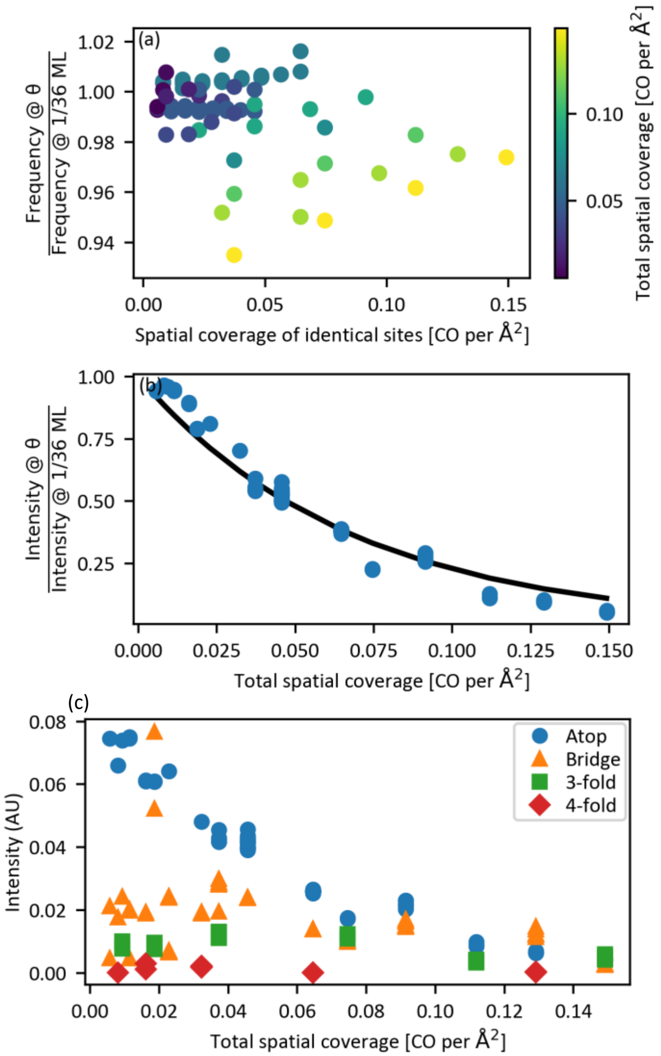

Supplementary Figure 2 | Scaling factors for Pt-CO normal modes at the atop binding site. Shown are the frequency (a) and intensity (b) scaling factors. Like the coverage scaling factors for the C-O modes, frequencies are regressed against both site-specific spatial coverage on the x-axis and total spatial coverage indicated in the color bar. (c) compares the intensities for Pt-CO modes at all binding-types correlated with total spatial coverage.

Supplementary Note 3: Quantifying Structure with PDFs

Binding-type pdf

The binding-type pdf corresponding to a given complex spectrum is produced by tabulating the individual binding-types corresponding to the constituent single spectra and assigning a percent of the complex spectra to each binding-type. Since binding-types are discrete numbers and there are only four of them, there are many data for each one. Furthermore, the binding-type itself captures underlying physics, as CO molecules adsorbed at atop and bridge sites have different hybridization with the surface.

Generalized coordination number (GCN) pdf

Physics is also embedded into the GCN values present in the primary dataset and how they cluster together in Figure 3 of the main text. Adsorption at some sites may be highly unfavorable (high energies if occupied), causing

the energy minimization algorithm in VASP to reduce the number of such sites in the primary dataset. The maximum GCN of atop binding that can accommodate an adsorbed CO is 8.5. Furthermore, symmetry of Pt nanoparticles results in lower energy structures and may be correlated with the ratio of under- to over-coordinated sites associated with given geometric motifs (octahedral, icosahedron, etc.). Note that small differences in GCN values would not result in detectible differences in spectra and the GCN value is slightly affected by the cutoff radius chosen (see Methods section of the main text).

Clustering algorithms provide an ideal way of assigning GCN ranges, each containing enough DFT data, which we refer to as a GCN group. Clustering is an established unsupervised learning method to classify data into groups, traditionally employed for pattern identification[10]. We use these groups to build GCN-pdfs for multinomial supervised regression. The GCN-pdf gives the percent of the complex spectra corresponding to CO adsorbing within certain GCN values. We use K-means clustering[11] to cluster GCN values representing the CO adsorption on nanoparticles into 9 groups and add a 10th group for the high coverage CO on low index-planes. This is a robust clustering algorithm that distributes data evenly into different GCN groups of approximately equal GCN ranges while minimizing the within-class variance[11]. Results of clustering at the occupied atop sites into GCN groups are given in Supplementary Figure 3.

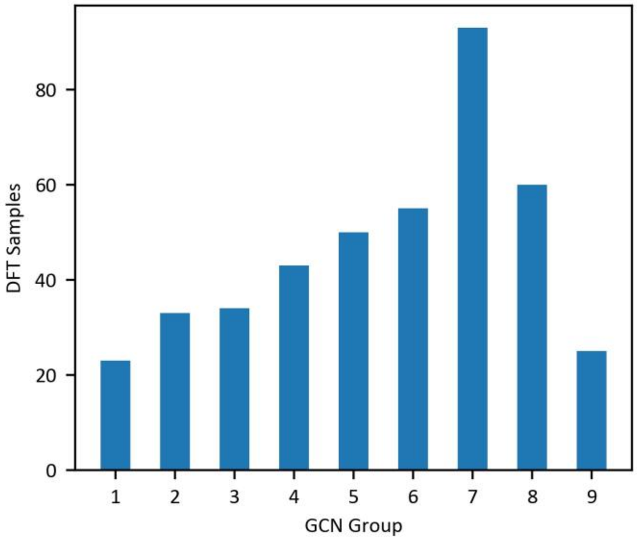

Supplementary Figure 3 | Binning of GCN values from primary zero-coverage data into GCN groups via k-means clustering. The [GCN group, GCN range] are as follows: [1, 0-1.8], [2, 1.8-2.8], [3, 2.8-3.7], [4, 3.7-4.5], [5, 4.5-5.2], [6, 5.2-6.1], [7, 6.1-7.0], [8, 7.0-7.9], [9, 7.9-8.5]. A 10th group indicates the high coverage/low-index planes ([10, high coverage]).

We develop GCN-pdfs for only atop-adsorbed CO because the spectra for this binding exhibits a distinct range in wavenumbers and there is a plurality of DFT and experimental data available, as this site tends to be the most favorable at both low and high coverage[1,4]. Contributions to the intensity from other binding-types (bridge, 3-fold, 4-fold) are added to the spectra from atop sites after generating the atop-site GCN pdfs.

Supplementary Note 4: Overview of Data Variability

When building data-based models, it is necessary that the input data used in training represents the desired predicted state space as proportionally as possible. For this reason we implement random oversampling of our primary training data to ensure that each binding-type or GCN range is represented equally. This procedure prevents overweighting of the majority class in model predictions. Because our model is trained on secondary data, we select the fraction of each binding-type or GCN range according to a probability distribution that allows sampling of the whole state space. Histograms of the resulting sample space for binding-type fraction and GCN-group fraction can be found in Supplementary Figure 4a and b, respectively. It is clear that small fractions are sampled more often than large fractions. This is a constraint imposed by having the model predict binding-types and GCN group fractions that sum to one (a probability distribution).

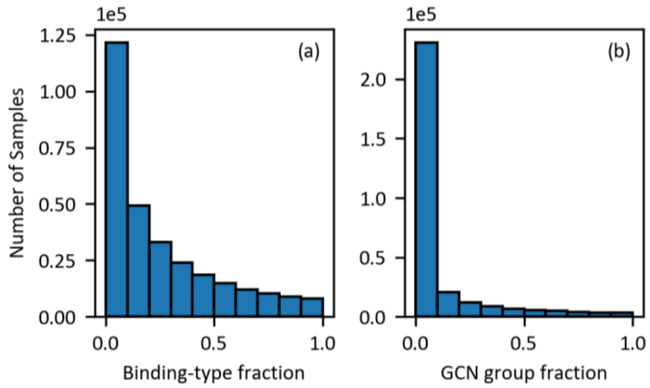

Supplementary Figure 4 | Probability density functions (pdfs) for synthetic data used in training, cross validation, and testing. Pdfs representing probability densities of fractional contribution of a generic binding-type (a), and a GCN group (b) from a set of 300,000 generated samples.

Supplementary Note 5: Learning Discrete PDFs with Multinomial Regression

To select a ML algorithm for mapping spectra to microstructure, we first tested several ML algorithms on mapping the primary DFT frequencies and intensities to binding-type and GCN. We found that a neural network performed well consistently. Furthermore, a neural network is also well suited for simultaneous regression of an ensemble of values (multinomial regression). We discuss the neural network in more detail in the next section.

The softmax output activation

We use the softmax as the output activation function for multinomial regression of discrete pdfs to regress the values of multiple variables simultaneously. The softmax squashes all of the output to be between zero and one with unit summation. The regressed output for each class (binding-type) or group (GCN group) is given by Supplementary Equation 1,

$$f_i = \frac{e^{a^T w_i}}{\sum_{k=1}^{K} e^{a^T w_k}}. \quad (1)$$

Here a is the vectorized output from the last hidden layer, w_i is the vector of weights for the ith group, and K is the group number to be regressed.

The Wasserstein loss function

Our GCN-pdf has 10 groups whose values must be regressed simultaneously. Complex multinomial regression requires a powerful loss function that can capture strong intergroup relationships. We choose the Wasserstein loss function,[12] as it improves learning stability in Generative Adversarial Networks[13], data generation and denoising through Boltzmann Machines[14], and multi-label classification with neural networks,[15] respectively, over kl-divergence and cross-entropy loss. For numerical purposes of computing the derivative, we use the Wasserstein loss squared (W) where

$$W = \sum_{n=1}^{C} \left[\sum_{i=1}^{n} p_i - \sum_{i=1}^{n} t_i \right]^2. \quad (2)$$

In Supplementary Equation 2, C is the number of groups in the pdf, p_i is the predicted probability value of the ith group, and t_i is the actual probability of the ith group.

Learning the parameters of a neural network through backpropagation requires a derivative of the loss function with respect to the output activation. To improve robustness and speed, we introduce an analytical derivative. Hou et al.[15] provides an open form solution for a general output activation function. For convenient vectorized

implementation, we introduce a closed form solution to the derivative of the Wasserstein squared specifically with respect to the softmax activation. The derivative (Supplementary Equation 3) is derived in the next section

$$\frac{\partial W}{\partial o_k} = 2p_k\left(\sum_{n=k}^{C}\left[\sum_{i=1}^{n}(p_i - t_i)\right] - \sum_{n=1}^{C}\left[\sum_{j=1}^{n}\left(p_j\sum_{i=1}^{n}[p_i - t_i]\right)\right]\right). \quad 3$$

In Supplementary Equation 3, o is equal to $a^T w$ of Supplementary Equation 1, k is the kth index of o, corresponding to the ith index of a discrete pdf formed upon transformation of o by the softmax. This closed form solution is general and can simultaneously learn discrete pdfs with any number of groups (see Methods for its vectorized form). The Wasserstein loss function can distinguish similarity between discrete distributions in a way that more common loss functions, such as mean squared error and KL-divergence, cannot. Note that the derivative is technically zero when one element in each probability being compared is exactly unity and is small when vectors being compared are far apart. This behavior of the derivative results in slow learning if the initial neural network parameters lead to predicted probabilities far from the actual ones. This slow learning can be compensated by a small addition of KL-divergence loss that gradually decreases as the neural network becomes more accurate. The closed form solution to the Wasserstein loss function, with respect to the softmax function, enables a neural network to perform multinomial regression and learn the corresponding binding-type- and GCN-pdfs.

Supplementary Note 6: Choice of Algorithm and Deriving the Wasserstein Derivative

We seek to predict two pdfs from given spectra. These pdfs are discrete distributions that represent the local structure of the nanoparticles and, at high coverage, the extended structure of the surfaces as well. While GCN is nearly continuous, we must discretize its pdf for it to be compatible with the both the neural network and the Wasserstein loss function. Furthermore, there is a trade-off between the detail and prediction accuracy. As more GCN groups are added to the discrete GCN-pdf, the similarities between those groups increase and the amount of DFT data in each group decreases leading to worse prediction accuracy. Most ML algorithms deal with either classification or regression of a single target variable. Even multi-class classification algorithms assign a single class out of many to the target based on a vector of features. Because we are interested in generating probability distributions, where the percent of each class must be regressed together all at once for a single prediction, we employ multinomial regression[16,17].

Choice of algorithm

We use a neural network because it is capable of generating any decision boundary with just a single layer given an infinite number of neurons[18]. Current implementations can be modified in a straightforward manner to allow multinomial regression. We also found that it performs at least as well as all other methods at classifying the binding-type and GCN group of a single site using the C-O and Pt-CO frequencies as input. Methods tested in this classification problem included decision trees, support vector machines, Gaussian process regression, and kernel ridge regression with various kernels. We implemented cross validation to identify the best models.

Derivative of the Wasserstein squared with respect to the softmax

To start, the generic derivative of W with respect to any output activation[15] is function is

$$\frac{\partial W}{\partial o_k} = 2\sum_{n=1}^{C}\left[\left(\sum_{i=1}^{n}\frac{\partial p_i}{\partial o_k}\right)\left(\sum_{i=1}^{n}p_i - \sum_{i=1}^{n}t_i\right)\right], \quad 4$$

where

$$o_k = a^T w_k. \quad 5$$

For the softmax function[19],

$$\frac{\partial p_i}{\partial o_k} = p_k(1-p_i), k = i \quad 6$$

and,

$$\frac{\partial p_i}{\partial o_k} = -p_k p_i, k \neq i. \quad 7$$

Writing this out for the simplest case where *C=2,* we have

$$\frac{\partial W}{\partial o_1} = 2[p_1(1-p_1)(p_1-t_1) + (p_1(1-p_1) - p_1p_2)(p_1+p_2-t_1-t_2)] \quad 8$$

and

$$\frac{\partial W}{\partial o_2} = 2[-p_2p_1(p_1-t_1) + (-p_2p_1 + p_2(1-p_2))(p_1+p_2-t_1-t_2)]. \quad 9$$

Rearrangement then gives us

$$\frac{\partial W}{\partial o_1} = 2\mathrm{p}_1\big((\mathrm{p}_1-t_1) + (p_1-t_1) + (p_2-t_2) - p_1[(p_1-t_1) + (p_1-t_1) + (p_2-t_2)] - p_2[(p_1-t_1) + (p_2-t_2)]\big) \quad 10$$

and

$$\frac{\partial W}{\partial o_2} = 2\mathrm{p}_2\big((p_1-t_1) + (p_2-t_2) - p_1[(p_1-t_1) + (p_1-t_1) + (p_2-t_2)] - p_2[(p_1-t_1) + (p_2-t_2)]\big). \quad 11$$

A closed form solution for any value of *C* is then seen visually as

$$\frac{\partial W}{\partial o_k} = 2p_k\left(\sum_{n=k}^{C}\left[\sum_{i=1}^{n}(p_i-t_i)\right] - \sum_{n=1}^{C}\left[\sum_{j=1}^{n}\left(p_j\sum_{i=1}^{n}[p_i-t_i]\right)\right]\right). \quad 12$$

A straightforward implementation of Supplementary Equation 12 in vectorized form is then

$$\frac{\partial W}{\partial O} = 2P\left(cumsum(cumsum(P-T)[::-1])[::-1] - sum\big(cumsum(P) * cumsum(P-T)\big)\right) \quad 13$$

where *∂W/∂O* is a vector of derivatives *∂W/∂o_k*, *P* is a predicted pdf vector, *T* is the actual pdf vector, *cumsum()* returns a cumulative summation vector, *sum()* returns a summation scalar, and [::-1] inverts a vector such that [1,2,…n-1,n][::-1]=[n,n-1,…2,1].

An example of the Wasserstein loss and its derivative with respect to the softmax output activation function is illustrated in Supplementary Figure 5. The loss and derivatives are computed for three simple vectors: [a,0,0,1-a] (green lines), [0,a,0,1-a] (blue lines), and [0,0,a,1-a] (orange lines) with vector [0,0,0,1]. Shown for comparison is the common KL-divergence loss and derivative with respect to the softmax (black lines).

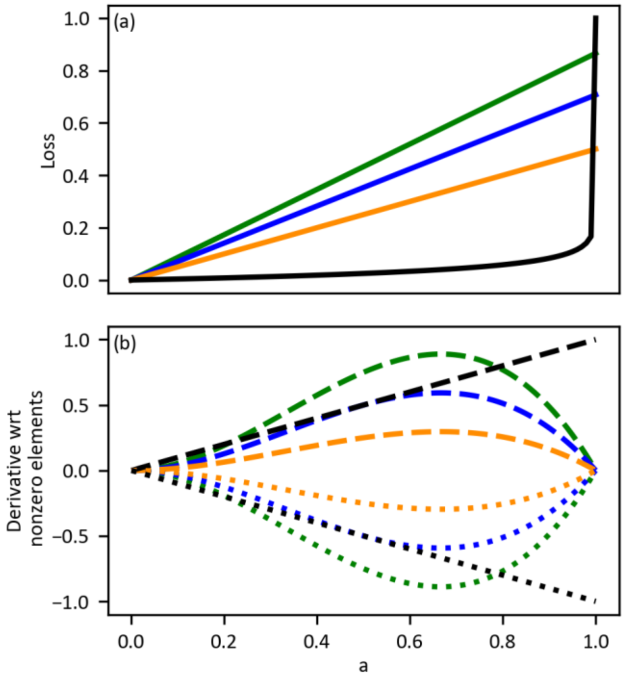

Supplementary Figure 5 | Wasserstein loss and its derivatives with respect to the softmax. The loss (a) and derivatives (b) are calculated for vectors [a,0,0,1-a] (green), [0,a,0,0,1-a] (blue), and [0,0,a,1-a] (orange) with vector [0,0,0,1] vs a. The loss is indicated by solid lines. The derivatives with respect to the indices with variable a and (1-a) are illustrated with dashed and dotted lines, respectively. Also shown is the KL-divergence loss and derivatives for all three vectors (black) with [0,0,0,1]. Note that the KL-divergence, a popular loss metric for probability distributions, cannot distinguish the loss nor the derivatives between any of the three vectors with variable a.

Supplementary Note 7: Hyperparameter Tuning and Model Assessment

The filtered DFT data is split into two datasets using stratification. 25% of the data (the hold-out set) is reserved for testing, while the remaining 75% (the training set) is selected for training and cross validation to identify the best parameters for the respective neural network models[20]. Each of these datasets is used to generate hundreds of thousands complex spectra. Primary data (DFT calculated frequencies and intensities) is split for training and testing/validation before generating the secondary data (synthetic spectra). Hyperparameter tuning was achieved using 3-fold cross validation to estimate model error on the same segmentations of the data. The neural network models with the lowest error were selected[20] for training using the Adam's algorithm with various segmentations of the data and included in an ensemble for predicting each pdf type. Comparison against the hold-out test set is used to get a final estimate of model error. A randomized search of hyperparameter space is performed with most of the parameter ranges given in Supplementary Table 4 and the rest in the text following the table.

Supplementary Table 4 | Initial hyperparameter space exploration. Initial hyperparameter space explored for selecting the neural network models to include in each type of ensemble model with unique ensemble models for each combination of adsorbate (CO, and NO), surface coordination pdf (binding-type, binding-type with all hollow sites considered one site, and GCN group), and coverage (high, low, or a numeric).

	Number Hidden Layers	Nodes per layer	Regularization parameter	Regularization Loss (norm)	Initial learning rate	Epsilon
Low Value	2	50	10^{-6}	L1	10^{-4}	10^{-14}
High Value	3	151	0.1	L2	10^{-3}	10^{-10}

For most of the models, the number of training points per training set varied between 5,000 and 50,000 with the number of training sets between 100 and 1,000 and the batch size between 10 and 1,000. Hyperparameter values used in training the neural networks included in the ensemble models are given in Supplementary Table 5 and the following text.

Supplementary Table 5 | Hyperparameters used in ensemble training. Hyperparameters used for training the neural network models to include in each type of ensemble model.

	Hidden Layer Structure	Regularization parameter	Regularization Loss (norm)	Initial learning rate	Epsilon	Batch size	Epochs per training set
Value sets	(100, 100, 100), (50, 50)	10^{-3} or $5x10^{-5}$	L1	0.0004	10^{-12}	50	10
Value sets	(100, 100, 100), (50, 50)	10^{-3} or $5x10^{-5}$	L1	0.0002	10^{-12}	10	10
Value sets	(75, 75, 75), (75, 75)	10^{-5}	L1	0.0004	10^{-12}	100	3

Neural networks included in the ensemble models for predicting GCN pdfs or binding-type pdfs at low or a single value of coverage were trained on 200 sets of training data with 5,000 complex spectra per training set except for the models trained using 3 epochs per training set which used 400 sets of training data.

Due to the long time to generate sets of synthetic spectra for high coverage systems, models trained to predict binding-type pdfs at high coverage had the number of training points per training set and the number of training sets fixed to 500 and 100, respectively, with a batch size of 10 for initial hyperparameter tuning. For all models the number of epochs per training set was 5. Hyperparameters selected for training neural networks used in the high coverage binding-type ensemble models were the same as those outlined in Supplementary Table 5 except the batch size was either 5 or 10, the number of trainings were 100, 200, or 300 with 500 complex spectra in each training set and the number of epochs was either 5, 20, 40.

An example of synthetic spectra and predicted binding-type pdf by one of the neural networks in the ensemble model can be found in Supplementary Figure 6. In Supplementary Figure 6a, the peaks near 2100 cm^{-1}, 1850 cm^{-1}, and 1700 cm^{-1} correspond to contributions from occupied atop, bridge, and hollow sites, respectively. The exact contributions of these sites as well as the contributions predicted by the model are given in Supplementary Figure 6b by the green bars with slanted lines and the purple bars with horizontal lines, respectively.

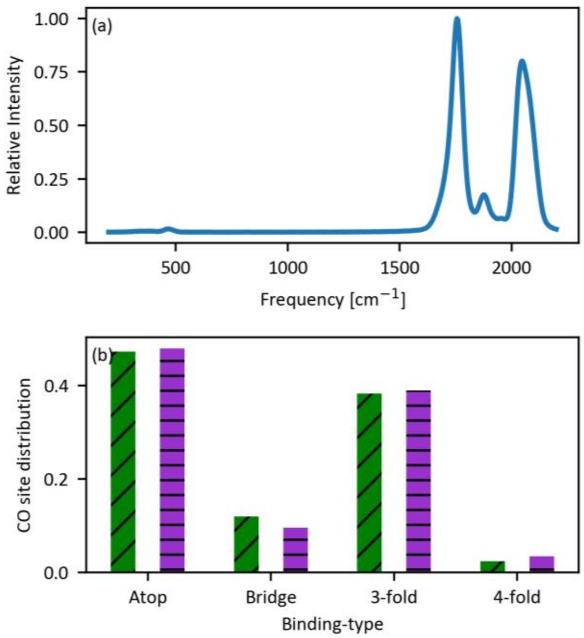

Supplementary Figure 6 | Synthetic complex spectra at high CO coverage. Depicted in (a) is the relative intensity against frequency using the methods outlined in this paper. In (b) is the corresponding distribution of CO contribution to the spectra from each binding-type. Shown are both the actual distribution (green bars with slanted stripes) and the pdf predicted by the binding-type surrogate model (purple bars with horizontal stripes).

Both R^2 and RMSE in the binding-type model are adequate measures of error because there are 4 distinct binding-types with large separation between frequencies and intensities. The RMSE for the GCN model is not however adequate due to the larger number of groups and the differences between neighboring groups being less distinct. For this reason, we use the Wasserstein loss score. The learning curves of both the binding- and GCN-pdf models in Supplementary Figure 7 reveals fast convergence in backpropagation when the derivative of the Wasserstein loss is used. Learning curves for just one of the neural networks in the ensemble for each type of structure model are shown. Both the mean cross validation and training loss (solid green and blue lines) are shown as well as the standard deviation of validation and training loss (dashed green and blue lines). Supplementary Figure 7c reveals that a better low coverage GCN model could be produced if it were trained longer as validation loss appears to still be decreasing.

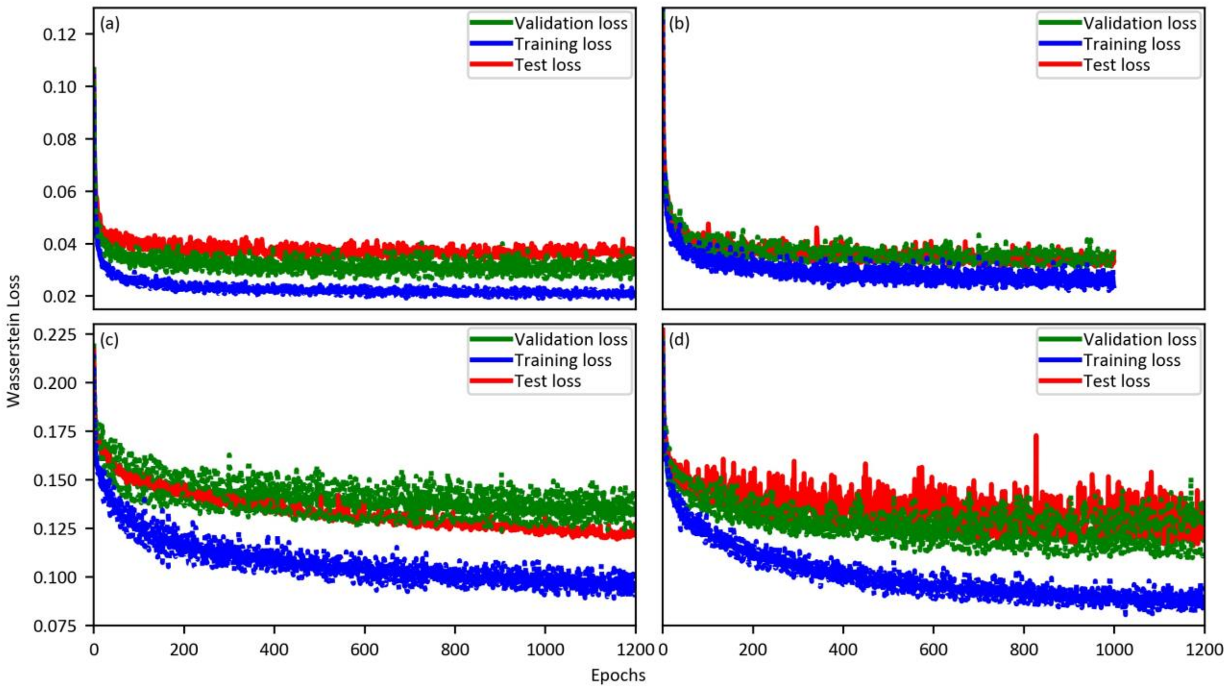

Supplementary Figure 7 | Learning curves for the best CO binding-type models at various coverages. Shown are binding-type models for low coverage (a) and high coverage (b) as well as the best GCN models at low coverage (c) and high coverage (d). Plotted is the loss vs. the number of epochs with 5,000 samples at a time, 3 epochs per training set and a batch size of 100 using the Adam's minimization algorithm except for figure (b) which used 500 samples at a time, 5 epochs per training set, and a batch size of 10. L1 regularization with a parameter of 10^{-5} was used in all cases. Shown is the training loss (blue line), validation loss (green line), and test loss (red line) along with the standard deviation in training loss (blue dotted) and validation loss (green dotted) line.

Two hundred randomly picked samples of binding-type model predictions on the hold-out test set of synthetic spectra from Supplementary Figure 7b are illustrated in Supplementary Figure 8. Shown are both the actual (x-axis) and predicted (y-axis) occupation of atop (blue circles), bridge (orange squares), 3-fold hollow (green triangles), and 4-fold hollow (red diamonds) sites as a percentage of the total occupied sites by CO. The error of the hollow sites is primarily due to ambiguity regarding which hollow sites are contributing to the spectra. Figure 3 in the main text shows that the greatest overlap of the spectra for any individual adsorption site occurs between 3-fold and 4-fold sites.

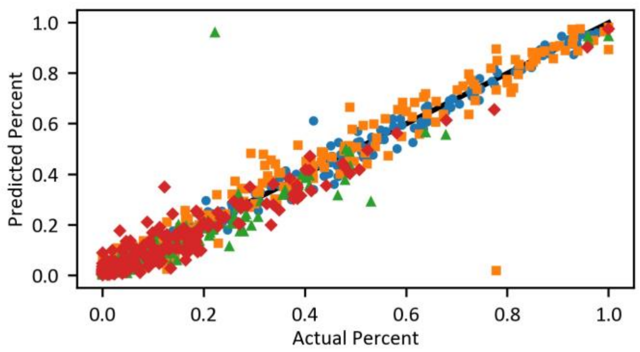

Supplementary Figure 8 | Assessment of high coverage CO binding-type model. Parity plot of predicted vs. actual binding-type pdfs for synthetic spectra of CO adsorption on Pt at high coverage. Atop (blue circles), bridge (orange squares), 3-fold (green triangles), and 4-fold (red diamonds) sites. RMSE for predicting each binding area are 0.033, 0.075, 0.066, and 0.047. The Wasserstein loss is 0.033.

In order to illustrate the effect of utilizing the low frequency wavenumbers, we show in Supplementary Figure 9 and Supplementary Figure 10 identical results to those in Supplementary Figure 7 and Supplementary Figure 8 except that only intensities corresponding to frequencies above 1000 cm^{-1} are used as descriptors by the neural networks. All errors for predicting low coverage and high coverage binding-type pdfs barely change but the errors increase significantly in predicted GCN pdfs. This signifies that, while the C-O frequency is nearly sufficient for

identifying whether CO is adsorbed to an atop, bridge, or hollow site, including lower frequency spectral descriptors significantly improves the predictive power for quantifying the coordination environment of the adsorption sites.

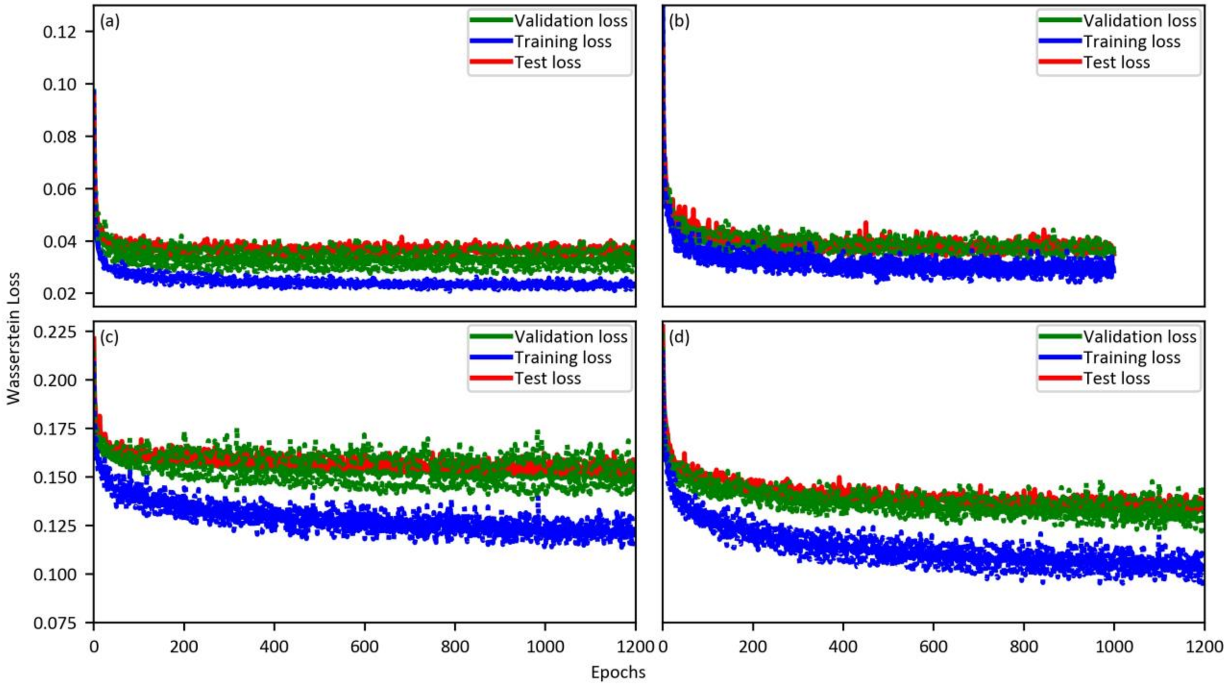

Supplementary Figure 9 | Learning curves for the best CO binding-type models at various coverages trained on spectra that excludes intensity below 1000 cm^{-1}. Shown are learning curves for binding-type models at low coverage (a) and high coverage (b) as well as the best GCN models at low coverage (c) and high coverage (d). Plotted is the loss vs. the number of epochs with 5,000 samples at a time, 3 epochs per training set and a batch size of 100 using the Adam's minimization algorithm except for figure (b) which used 500 samples at a time, 5 epochs per training set, and a batch size of 10. L1 regularization with a parameter of 10^{-5} was used in all cases. Shown is the training loss (blue line), validation loss (green line), and test loss (red line) along with the standard deviation in training loss (blue dotted) and validation loss (green dotted) line.

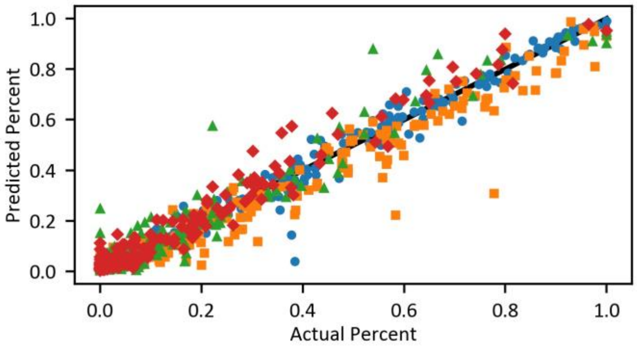

Supplementary Figure 10 | Assessment of high coverage CO binding-type model. Parity plot of predicted vs. actual binding-type pdfs for synthetic spectra of CO adsorption on Pt at high coverage. Atop (blue circles), bridge (orange squares), 3-fold (green triangles), and 4-fold (red diamonds) sites. RMSE for predicting each binding area are 0.046, 0.075, 0.059, and 0.047. The Wasserstein loss is 0.037.

Supplementary Note 8: Explanation of Experimental Spectra

Experimental spectra for CO with known ordered overlayers on Pt facets and on an Au/Pt core/shell nanoparticle are given in Supplementary Figure 11. Combined LEED and TPD measurements revealed that at 0.5 ML on a Pt(111) surface, the CO is in a c(4x2) configuration[1,21] with 50% of the CO adsorbed at atop sites and the rest at bridge sites[21]. At higher coverages[22], the spectra corresponds to 62% atop and 38% bridge sites, assuming that all additional CO on Pt(111) beyond 0.5 ML adsorbs at atop sites. The same LEED studies suggest that nearly all of the CO is at atop sites below 0.18 ML[21]. LEED and DFT combined suggest that, although the Pt(110) surface undergoes reconstruction, at 1ML of CO the surface deconstructs with Pt in p(2x1) configuration and all of the exposed atop sites occupied[23]. Although LEED was not performed on the more complex Au/Pt core shell nanoparticles, STM reveals the nanoparticles have little variation in their structure and are highly symmetric[24], which would suggest most of the Pt surface consists of low-index planes. Due to relatively low solubility of CO in H_2SO_4, we expect the surface coverage of CO on the nanoparticles to be low.

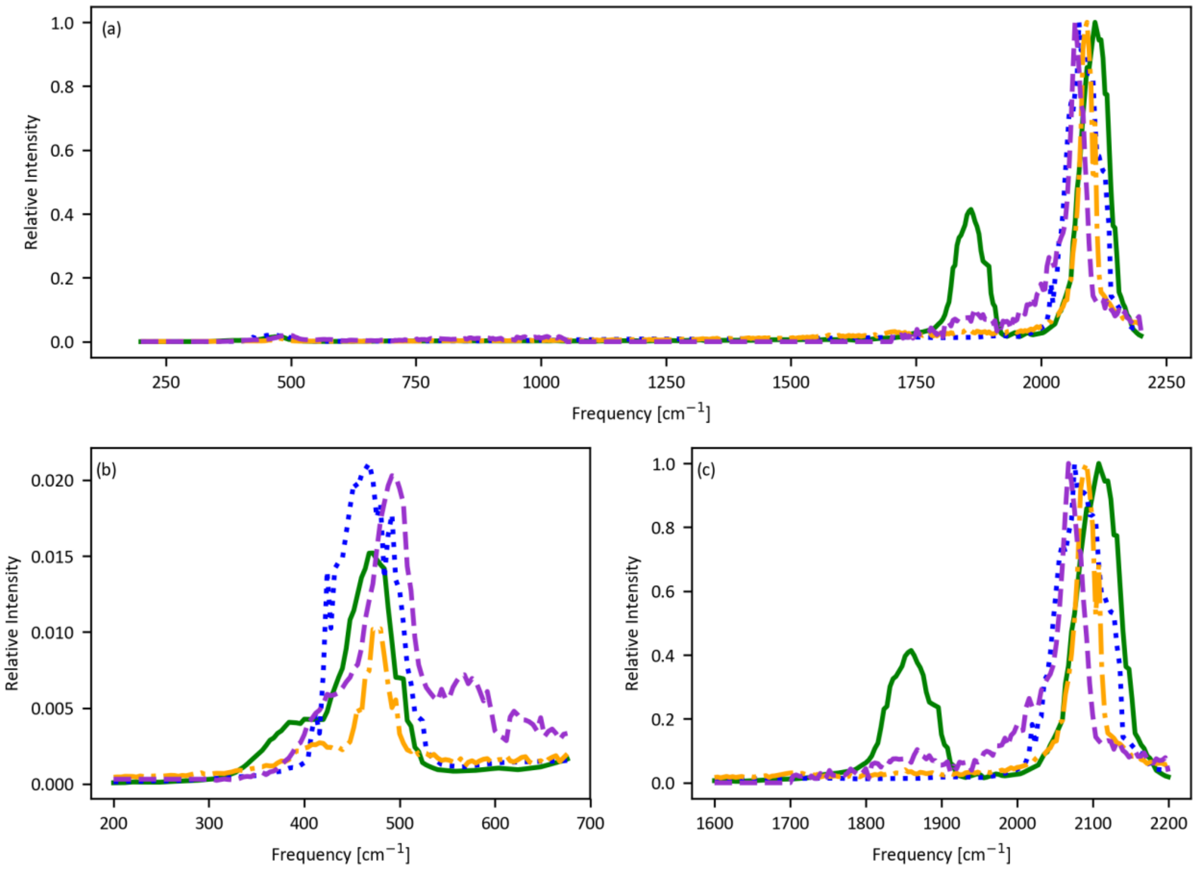

Supplementary Figure 11 | Experimental HREEL and SERS spectra for CO on platinum scaled by wavenumber to the power of 2.7 to convert to IR spectra. Subplots reveal the whole spectra (a), the Pt-CO stretch (b), and the C-O stretch (c). In each subplot is CO on Pt(111) in a c(4x2) configuration at 0.5 ML (green line), at 0.17 ML on a Pt(111) surface (blue dotted line), on a Pt(111) surface at 1 ML (yellow dashed dotted line), and low coverage on Au/Pt core/shell nanoparticles (purple dashed line). Shown is normalized intensity (y-axes) vs. wavenumber (x-axes).

Frequency overlap of CO adsorbed at atop sites at 0.5 ML total coverage (green solid line) and 0.17 ML total coverage (blue dotted line) on the Pt(111) surface in the Pt-CO stretch region is present (Supplementary Figure 11b) and less present in the C-O stretch region (Supplementary Figure 11c). Spectra corresponding to CO on nanoparticles (purple dashed lines) overlap with spectra from CO at 0.17 ML coverage in the C-O stretch region but not in the Pt-CO stretch region. It is evident that it is difficult to distinguish the CO coverage using its spectra without aid of

advanced regression tools. To further illustrate this point, Supplementary Figure 12 depicts C-O (a) and Pt-CO frequencies (b), as well as their intensities, for CO adsorbed on atop and bridge sites with a generalized coordination number of approximately 7.5. These nanoparticle sites therefore emulate the (111) extended surface and yet ratios of atop and bridge intensities differ from those of the extended surface (black). This is the primary source of uncertainty in the predictions depicted in Figure 6b of the main text.

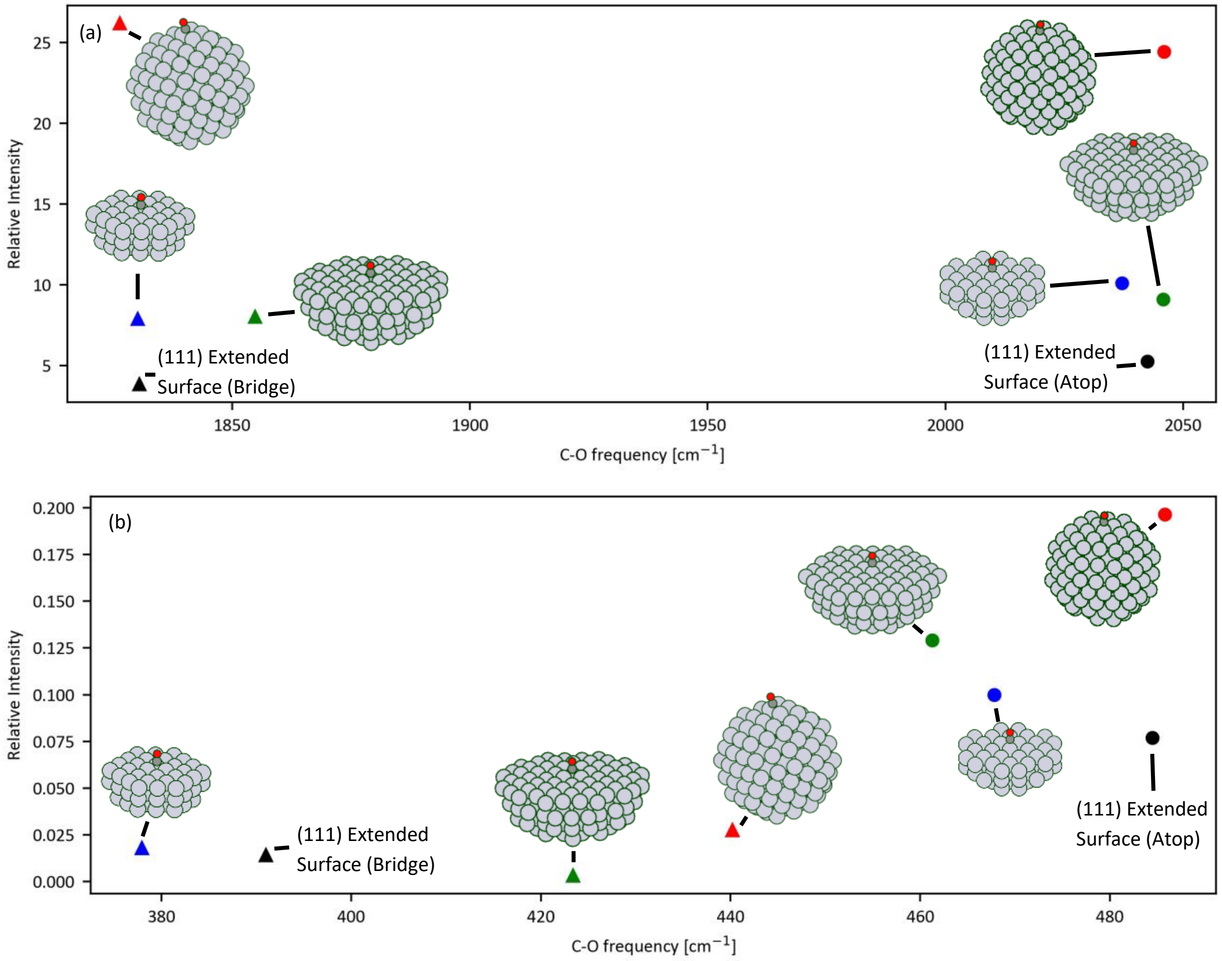

Supplementary Figure 12 | Different nanoparticle adsorption sites that approximate a (111) surface. Shown are DFT calculated frequencies and intensities for adsorption at atop sites (circles) bridge sites (triangles) of identical nanoparticles as well as for the extended surface. Frequencies and intensities correspond to the C-O (a) and Pt-CO (b) stretch vibrations. Even for large nanoparticles consisting of 200 Pt atoms, the corresponding spectra are far from that of the extended surface.

Supplementary Note 9: Details of First-Principles Calculations

The DFT setup is provided in the Methods section. Supplementary Figure 13 demonstrates that non-spin polarized calculations without dipole corrections are adequate for generating our DFT nanoparticle data. While energies were greatly affected by the introduction of spin-polarization for small nanoparticles, frequencies and intensities of adsorbates were not. This fact allows us to save significant computational time, especially for large nanoparticles. We note that all systems with extended surfaces, as well as with NO, which are discussed in the next section, included dipole corrections. Systems smaller than 60 atoms with NO included spin polarization.

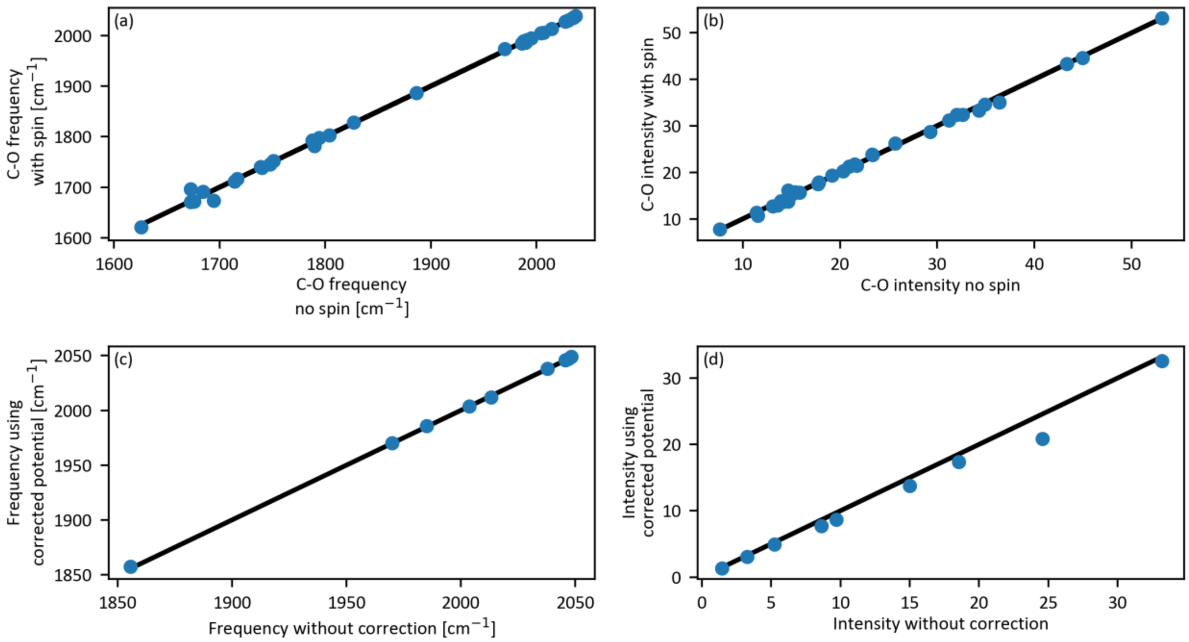

Supplementary Figure 13 | Parity plots assessing the impact of spin polarization and image dipole corrections on frequencies and intensities of CO adsorbed on Pt nanoparticles. Spin polarization has a minor effect on frequencies (a) and intensities (b)**.** Image dipole for carbon monoxide on Pt nanoparticles also alters frequencies (c) and intensities (d) only minimally.

We also calculate adsorption energies for 77 unique adsorption sites on 17 unique nanoparticles with full relaxation to illustrate that C-O frequency does not correlate with CO adsorption energy.

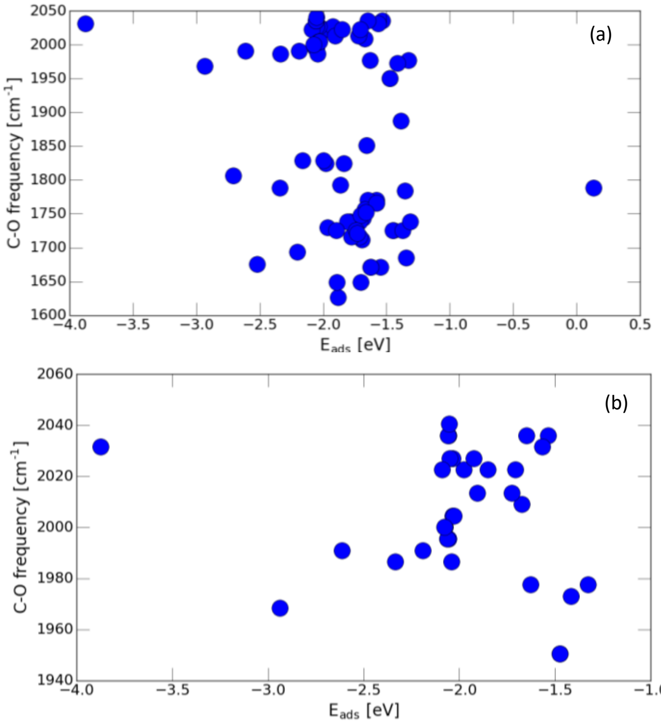

Supplementary Figure 14 | C-O frequency vs. adsorption energy (E_{ads}) on 77 unique adsorption sites of 17 unique nanoparticles. Shown is data for all binding-types (a) and only atop sites (b).

Supplementary Note 10: Extension with NO as a Probe Molecule

We visualize the primary dataset for NO frequencies and intensities in Supplementary Figure 15 in an identical manner to those for CO in Figure 3 of the main text. The primary dataset includes NO at atop (green circles), bridge (red squares), 3-fold (yellow triangles), and 4-fold (blue diamonds) adsorption sites. After removing outliers from the primary NO dataset, the resulting filtered primary dataset consists of 653 local minima (Supplementary Figure 15b). Supplementary Figure 15c and d show the Pt-NO frequency and N-O intensity, respectively, against the N-O frequency with the GCN value indicated in the color bar. Improved separation of data with similar GCN values is visible, although less so than with CO. The scaling of N-O intensities with total spatial coverage is shown in Figure 5b of the main text.

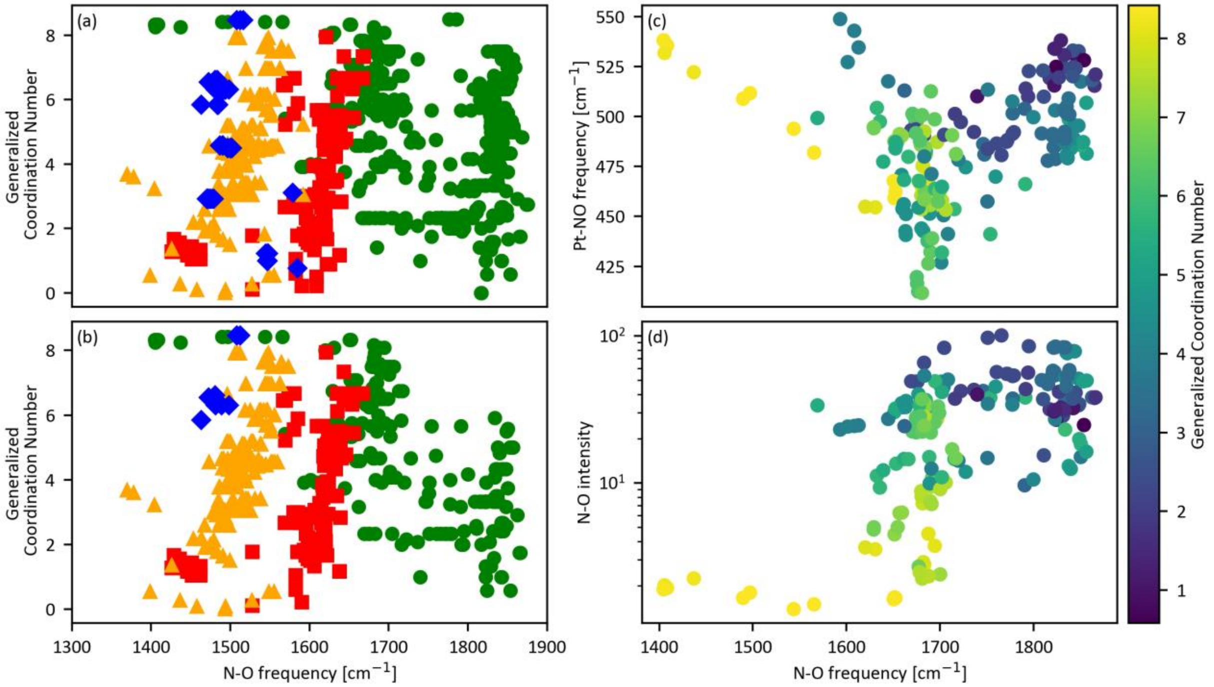

Supplementary Figure 15 | Visualization of the primary DFT dataset and removal of outliers for NO on Pt nanoparticles. a-d, Data visualized in terms of the generalized coordination number (GCN), N-O frequencies, and/or intensities calculated for chemisorbed NO. a, primary data set. b, dataset after removing outliers (see text). Colors/shapes indicate GCN and frequency pairs for NO chemisorbed on atop (green/circles), bridge (red/squares), 3-fold (yellow/triangles) and 4-fold (blue/square) sites. c,d, 2D visualizations of chemisorbed Pt-NO stretch frequencies and intensities, respectively, at atop sites vs. the N-O stretch frequency. The color of the points indicates the value of the GCN depicted in the color map on the right. Colors go from low (violet) to high (yellow) values.

As is the case with CO, DFT calculations at different total spatial coverages on 111, 100, and 110 low-index planes of NO at the atop (circles), bridge (squares), 3-fold (triangles) and 4-fold (diamonds) sites reveal universal linear scaling of N-O (Supplementary Figure 15). The coverage scalings are universal because data points from different extended surfaces at sites with different GCN values fall on the same line. Coefficients regressed on data in Supplementary Figure 15 in Equation 3 of the main text using OLS on high coverage, extended-surface DFT data and associated R^2 values are shown in Supplementary Table 6. Standard errors in the regressed parameters are also given in the table. Details regarding calculations are the same as those for CO coverage scaling factors already described. R^2 values for the N-O coverage scaling factors are slightly lower than for CO.

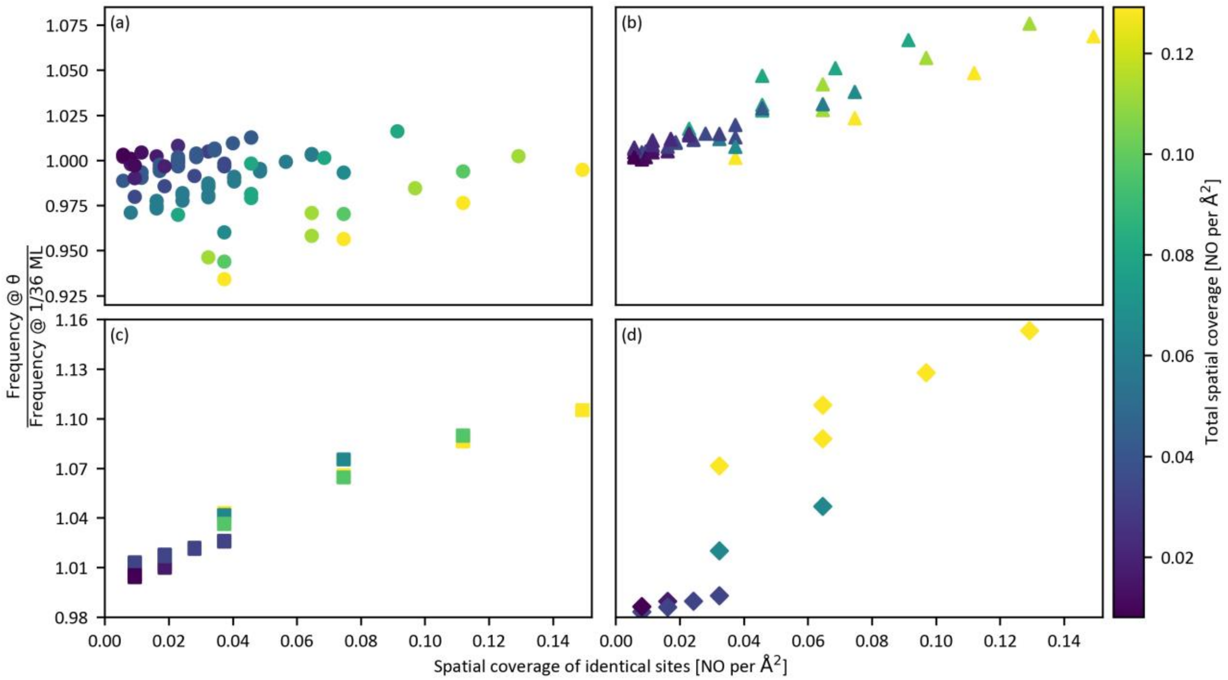

Supplementary Figure 16 | NO coverage-IR linear scaling relations. Frequency-coverage scaling factors (y-axis) for atop (a), bridge (b), 3-fold (c) and 4-fold (d) hollow sites. Data is regressed using ordinary least squares (OLS) on both site-specific spatial coverage (x-axis) and total spatial coverage (color variation) of NO. The R^2 values of the regression are 0.793 (a), 0.931 (b), 0.988 (c), and 0.924 (d). Replacing site-specific spatial coverage with site-specific relative coverage results in better statistical models for the atop and bridge bound species; however, using only spatial coverage in the model allows for enforcing spatial coverage constraints when generating the secondary dataset of synthetic spectra. Data from the 111, 100, and 110 surfaces fall on the same line for each binding-type at either constant total spatial coverage or spatial coverage of identical sites.

Supplementary Table 6 | NO coverage scaling factor (CSF) regression coefficients. Coefficients for scaling frequencies from zero to elevated coverage. Coefficients a_{ss} multiply spatial coverage of similar sites and coefficients a_T multiply total spatial coverage.

Binding-Type	a_{ss} [Å^2 per CO]	$\sigma(a_{ss})$	a_T [Å^2 per CO]	$\sigma(a_T)$	R^2
Atop	0.539	0.049	-0.501	0.032	0.793
Bridge	0.592	0.051	-0.042	0.034	0.931
3-fold	0.627	0.046	0.137	0.033	0.988
4-fold	0.693	0.263	0.377	0.176	0.924

Although Pt-NO frequencies do shift with total spatial coverage, unlike the case with CO, Pt-NO frequencies do not shift with spatial coverage of identical sites given a constant total spatial coverage (see Supplementary Figure 17a). Furthermore, the intensities of the Pt-NO frequencies at atop sites (Supplementary Figure 17b) do not appear to scale with total spatial coverage in the systematic way that Pt-CO frequencies do (Supplementary Figure 2b). This difference could be due to numerical errors driven by the extremely low Pt-NO atop intensities (Supplementary Figure 17c; blue circles).

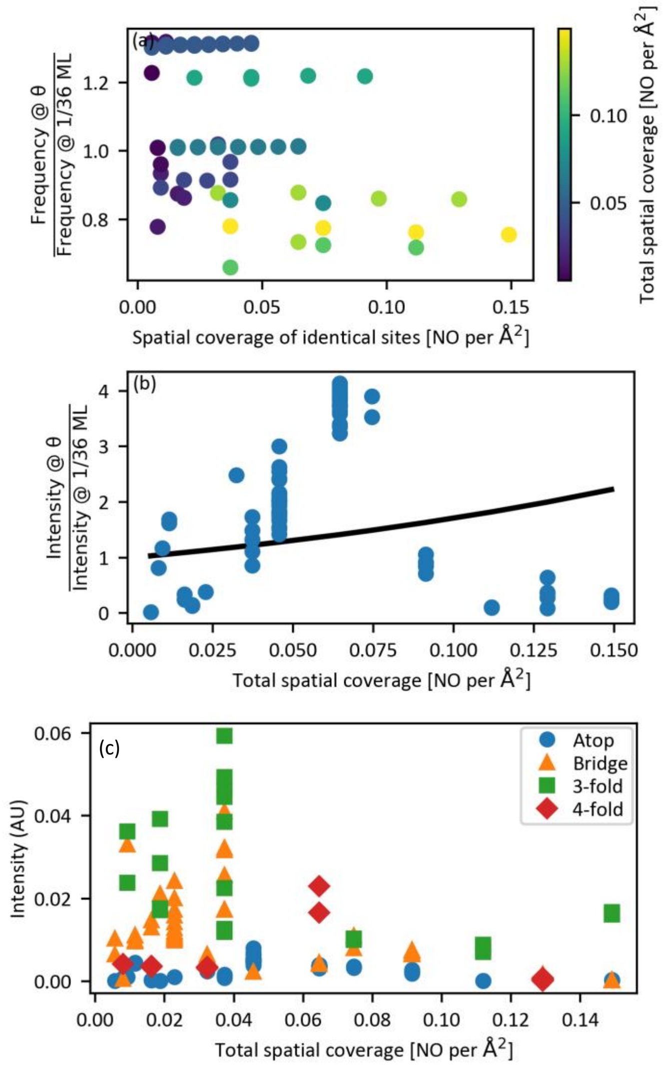

Supplementary Figure 17 | Scaling factors for Pt-NO normal modes at the atop binding site. Shown are the frequency (a) and intensity (b) scaling factors. Like the coverage scaling factors for the C-O modes, frequencies are regressed against both site-specific spatial coverage on the x-axis and total spatial coverage indicated in the color bar. (c) compares the intensities for Pt-NO modes at all binding-types correlated with total spatial coverage.

Learning curves for just one of the neural networks in the ensembles for four types of NO based structure model are shown in Supplementary Figure 18. Both the mean cross validation and training loss (solid green and blue lines) are shown as well as the standard deviation of validation and training loss (dashed green and blue lines). There is a much larger gap between the training and validation/test error with the models for NO than with CO. This likely results the fact there is less NO data.

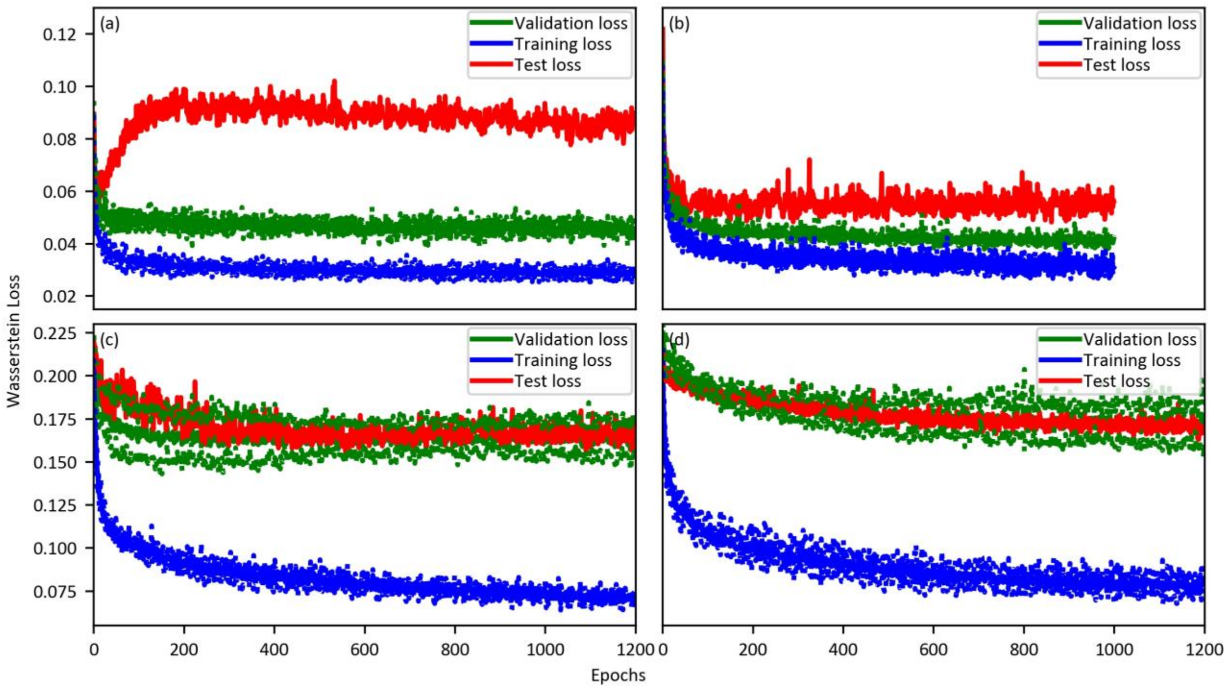

Supplementary Figure 18 | Learning curves for the best NO binding-type models at various coverages. Shown are learning curves where hollow sites are considered one group with a binding-type model at a coverage of 1 ML (a) and high coverage (b) as well as the best GCN models at a coverage of 1 ML (c) and low coverage (d). Plotted is the loss vs. the number of epochs with 5,000 samples at a time, 3 epochs per training set and a batch size of 100 using the Adam's minimization algorithm except for figure (b) which used 500 samples at a time, 5 epochs per training set, and a batch size of 10. L1 regularization with a parameter of 10^{-5} was used in all cases. Shown is the training loss (blue line), validation loss (green line), and test loss (red line) along with the standard deviation in training loss (blue dotted) and validation loss (green dotted) line.

Two hundred randomly picked samples of binding-type model predictions on the hold-out test set of synthetic spectra where hollow sites are combined into one binding-type with loss curve in Supplementary Figure 18b are illustrated in Supplementary Figure 19. Shown are both the actual (x-axis) and predicted (y-axis) occupation of atop (blue circles), bridge (orange squares), 3/4-fold hollow (green triangles sites as a percentage of the total occupied sites by NO). Even though hollow sites are combined, the error is still greater than that for the binding-type model where CO is the probe molecule.

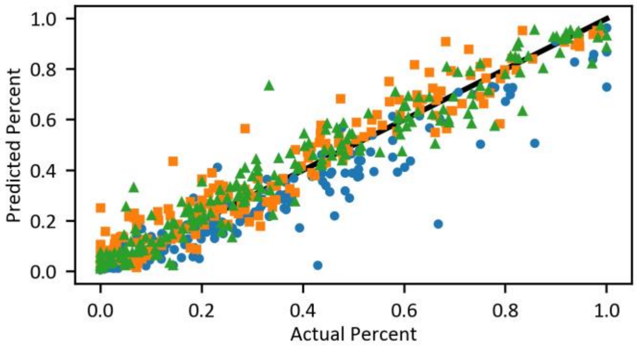

Supplementary Figure 19 | Assessment of high coverage NO binding-type model with combined hollow sites. Parity plot of predicted vs. actual binding-type pdfs for synthetic spectra of NO adsorption on Pt at high coverage. Atop (blue circles), bridge (orange squares), 3/4-fold (green triangles).

Supplementary Note 11: Step by Step Instructions for the Spectra to Structure Software

Here we provide detailed instructions for using our existing trained models on one's experimental spectra as well as step by step instructions for using our programming package to train and implement an ensemble of one's neural networks using one's DFT data. All code for the package used in generating the complex synthetic spectra, training the neural networks, and generating figures in the manuscript can be found in

https://github.com/VlachosGroup/jl_spectra_2_structure. All documentation can be found at https://vlachosgroup.github.io/jl_spectra_2_structure/. A zip folder of all relevant VASP calculations and pre-trained neural networks is stored on Zenodo at https://zenodo.org/record/3666992#.XkbVTGhKiCi.

1) Copy the contents from https://github.com/JLans/jl_spectra_2_structure onto your computer
2) Switch your working directory to the working directory that contains the jl_spectra_2_structure setup.py file and type `python setup.py`. All dependencies will be install automatically.
3) Download zip folder of VASP, CHARGEMOL, and trained neural networks from Zenodo.

Using existing trained neural networks or neural network ensembles

If one wants to use pre-trained neural networks or neural network ensembles, one can navigate to the directory from Zenodo titled "Lansford – Nature Communications 2020". Inside this directory is another directory labeled cv_BW. Folders titled "CO_BINDING_TYPE_HIGH", "CO_GCN_HIGH, and "learning_curves" contain 3-fold cross validation simulation runs on the Blue Waters Super Computer that have roughly equal minimum cross validation loss for each adsorbate and coverage combination. For each set of hyperparameters there are 5 cross validation runs with different segmentations of primary DFT data used in generating the secondary data (complex spectra) for training, validation, and testing. The data from these runs are stored in files that have unique file names and end in .json. These .json files contain the trained neural network coefficients and bias parameters as well. There are pre-trained neural networks for the following systems: CO binding-type on Pt nanoparticles at low and high coverage and at 1ML, CO GCN group on Pt nanoparticles at low coverage, high coverage, and at 1 ML, NO binding-type on Pt nanoparticles at low and high coverage and at 1 ML, NO GCN group on Pt nanoparticles at low and 1ML coverage.

1) To use a neural network ensemble, one selects .json files for the same physical system (i.e., the same adsorbate, target (binding-type or GCN), and coverage and place them into a folder.
2) Navigate to the scripts folder and open Predict_Experiment.py.
3) In Predict_Experiment.py change the file paths assigned to variables "BINDING_TYPE_PATH" and "GCN_PATH" to your folder that contains the collection of .json files.
4) Replace "get_exp_data_path()" in line 29 with a list of .csv files representing the experimental data where the first column is wave numbers and the second column is intensities. Alternatively, before running the command `python setup.py` Your own .csv files containing experimental data can be saved in package data subfolder named "experimental". The user's experimental data will then become the default experimental data.
5) If one uses IR data instead of HREEL or Raman set input parameter PEAK_CONV to 0 on line 32.
6) Run the script by typing `python Predict_Experiment.py`.

More details about the functions and methods used in "Predict_Experiment.py" are available in the documentation website, especially the "Examples" page.

Generating one's own primary dataset

When collecting experimental infrared (IR), Raman, or electron energy loss spectroscopy, we suggest collecting spectra at several coverages in order to identify changes in the surface structure as coverage is increased. If spectra is collected operando, we suggest collecting data at variable flow rates. If structure does not change with coverage, then the predictions at various coverages can be combined to reduce uncertainty.

Here we cover using the user's own DFT calculations for the user's own combination of adsorbates and surfaces, generating a primary DFT dataset. All primary data is contained in the "data" folder which is installed as python package data. The files that contain the primary DFT data at low coverage are titled 'single_"adsorbate".json' where "adsorbate" is the name of the user's adsorbate and contains lists of frequencies, intensities, binding-types, and GCN groups. Primary data used in generating coverage scaling factors is saved in files names 'isotope_"adsorbate".json' and files that contain high coverage DFT primary data are named 'high_coverage_"adsorbate".json`.

1) Forces should be saved to vasprun2.xml files and charge data should be saved to charge2.extxyz respectively. See example data files from Zenodo. These are concatenated vasprun.xml and DDEC6 charge files obtained for single point VASP and CHARGEMOL calculations, respectively. These calculations correspond to displacements to parameterize a Hessian and Jacobian for computing IR frequencies and intensities, respectively. Methods in primary_data_creation.file_parser convert these concatenated files into charge2.extxyzstripped.extxyz and vasprun2.xmlstripped.xml files that can be read by PANDAS and ASE python packages, respectively.
2) Files that contain low coverage and high coverage of adsorbates on nanoparticle and extended surface transition metal surfaces should all be contained in separate folders. If non-electrically conductive or partially electrically conductive extended surfaces are to be studied, “directions=2” on line 281 of file dft_2_data.py should be removed.
3) The file that generates the primary data sets is located in the “scripts” directory and named “generate_primary_data.py”. Open this file.
4) Change lines 23 through 28 to the directories that contain first-principles calculations for adsorbates on nanoparticles and extended surfaces. If variable names are changed, edit any following lines accordingly.
5) Change ‘CO’ on line 37 to the user’s adsorbate and set the paths to the primary data files to the files created by the user
6) When creating an instance of Primary_DATA class, several variables need to be changed to fit your primary DFT data. Please read the online documentation regarding that class for details and limitations of each input variable.
7) This class has methods that can generate primary data for nanoparticles and extended surfaces as well as isotopic data used in generating coverage scaling factors. For more specific details please read the online documentation.
8) In order for the coverage scaling relations file to be generated, generate_isotope_data must first be run.
9) See get_sf_and_variance.py in the scripts folder for generating the scaling factors for frequencies that are over and under 1000 cm^{-1}. The output from this file is hard coded into methods “scaling_factor_shift” and “_perturb_and_shift” of the class IR_GEN.
10) To generate Figure 2 of the main text that contains visualizations of the primary data use script “visualize_primary_data.py” in the main script. To generate Figures 3 and 4 of the main text, use script “visualize_scaling_data.py”.

Training one’s own neural network and running cross validation trials

We provide a cross validation class that is used to run the cross validation trials. It can also be used to generate a single batch of secondary data from the primary data and train a neural network once.

1) Run script “run_cv.py” in the scripts folder to run a single set of cross validation simulations using the class CROSS_VALIDATION and method run_CV_multiprocess. If only a single neural network needs to be trained, method get_test_results should be uncommented. For more details please see online documentation.
2) In order to select the best neural network hyperparameters, hundreds of cross validation trials are necessary using the same set of primary data to generate secondary data for each validation fold and test set. Using different sets of primary data for different trials can cause differences in validation scores simply due to how the variance in the data is distributed. “generate_cv_indices.py” in the “scripts/cv_BW” folder partitions the indices and saves them into a “folder/file” that can be designated by the user. All cross validation trials can then partition the primary data according to the indices. The mpi script that executes the hundreds of cross validation trials is mpicommexecutor.py. The submission script for blue waters is titled mpi.pbs.

3) Once several sets of hyperparameters that produce similar cross validation errors are chosen, mpi_cv_many_runs.py can in the "scripts/cv_BW" folder can be used to run X cross validation trials for the same set of hyperparameters while shuffling the data differently each time.
4) Use load_cv_sets.py in the scripts folder to visualize which sets of hyperparameters yield the lowest accuracy. There is not a single solution.
5) Use load_many_NN.py to generate the learning curves in this supporting information where the trained neural networks are applied to synthetic secondary data in the validation and test sets.
6) After training the neural networks, refer to "Using existing trained neural networks or neural network ensembles" to apply the neural networks to experimental data.

Supplementary References

1 Steininger, H., Lehwald, S. & Ibach, H. On the adsorption of CO on Pt(111). *Surf. Sci.* **123**, 264-282, doi:http://dx.doi.org/10.1016/0039-6028(82)90328-4 (1982).

2 Schweizer, E., Persson, B. N. J., Tüshaus, M., Hoge, D. & Bradshaw, A. M. The potential energy surface, vibrational phase relaxation and the order-disorder transition in the adsorption system Pt{111}-CO. *Surf. Sci.* **213**, 49-89, doi:https://doi.org/10.1016/0039-6028(89)90252-5 (1989).

3 Kizhakevariam, N., Jiang, X. & Weaver, M. J. Infrared spectroscopy of model electrochemical interfaces in ultrahigh vacuum: The archetypical case of carbon monoxide/water coadsorption on Pt(111). *J. Chem. Phys.* **100**, 6750-6764, doi:https://doi.org/10.1063/1.467271 (1994).

4 Klünker, C., Balden, M., Lehwald, S. & Daum, W. CO stretching vibrations on Pt(111) and Pt(110) studied by sumfrequency generation. *Surf. Sci.* **360**, 104-111, doi:http://dx.doi.org/10.1016/0039-6028(96)00638-3 (1996).

5 Gardner, P., Martin, R., Tüshaus, M. & Bradshaw, A. The adsorbate-induced lifting of the Pt100 surface reconstruction: IRAS investigations. *J. Electron. Spectrosc. Relat. Phenom.* **54**, 619-628 (1990).

6 Martin, R., Gardner, P. & Bradshaw, A. M. The adsorbate-induced removal of the Pt{100} surface reconstruction. Part II: CO. *Surf. Sci.* **342**, 69-84, doi:https://doi.org/10.1016/0039-6028(95)00679-6 (1995).

7 Persson, B. N. J. & Ryberg, R. Vibrational line shapes of low-frequency adsorbate modes: CO on Pt(111). *Phys. Rev. B.* **40**, 10273-10281, doi:https://doi.org/10.1103/PhysRevB.40.10273 (1989).

8 Surman, M., Hagans, P. L., Wilson, N. E., Baily, C. J. & Russell, A. E. Adsorption of CO on Pt{111}: a synchrotron far-infrared RAIRS study. *Surf. Sci.* **511**, L303-L306, doi:https://doi.org/10.1016/S0039-6028(02)01565-0 (2002).

9 Feldt, C. D. *et al.* CO Adsorption on Au (332): Combined Infrared Spectroscopy and Density Functional Theory Study. *J. Phys. Chem. C* (2018).

10 Jain, A. K., Murty, M. N. & Flynn, P. J. Data clustering: a review. *ACM Comput. Surv.* **31**, 264-323 (1999).

11 MacQueen, J. in *Proceedings of the fifth Berkeley symposium on mathematical statistics and probability.* 281-297 (Oakland, CA, USA).

12 Levina, E. & Bickel, P. in *Proceedings Eighth IEEE International Conference on Computer Vision.* 251-256 vol.252.

13 Arjovsky, M., Chintala, S. & Bottou, L. in *International Conference on Machine Learning.* 214-223.

14 Montavon, G., Müller, K.-R. & Cuturi, M. in *Advances in Neural Information Processing Systems.* 3718-3726.

15 Hou, L., Yu, C.-P. & Samaras, D. Squared Earth Mover's Distance-based Loss for Training Deep Neural Networks. *arXiv preprint https://doi.org/arXiv:1611.05916* (2016).

16 McCullagh, P. Regression Models for Ordinal Data. *Journal of the Royal Statistical Society. Series B (Methodological)* **42**, 109-142 (1980).

17 Andresen, M. A. & Jenion, G. W. The Unspecified Temporal Criminal Event: What is Unknown is Known with Aoristic Analysis and Multinomial Logistic Regression. *Western Criminology Review* **5** (2004).

18 Stinchcombe & White. in *International 1989 Joint Conference on Neural Networks.* 613-617 vol.611.

19 Dunne, R. A. & Campbell, N. A. in *Proc. 8th Aust. Conf. on the Neural Networks, Melbourne.* 185 (Citeseer, 1997).

20 Kohavi, R. in *Proceedings of the 14th international joint conference on Artificial intelligence - Volume 2* 1137-1143 (Morgan Kaufmann Publishers Inc., Montreal, Quebec, Canada, 1995).

21 Hayden, B. E. An infra-red reflection absorption study of the adsorption of CO on Pt(111). *Surf. Sci.* **131**, 419-432, doi:http://dx.doi.org/10.1016/0039-6028(83)90287-X (1983).

22 Norton, P. R., Davies, J. A. & Jackman, T. E. Absolute coverages of CO and O on Pt(111); Comparison of saturation CO coverages on Pt(100), (110) and (111) surfaces. *Surf. Sci.* **122**, L593-L600, doi:https://doi.org/10.1016/0039-6028(82)90054-1 (1982).

23 Karakatsani, S., Ge, Q., Gladys, M. J., Held, G. & King, D. A. Coverage-dependent molecular tilt of carbon monoxide chemisorbed on Pt{110}: A combined LEED and DFT structural analysis. *Surf. Sci.* **606**, 383-393, doi:https://doi.org/10.1016/j.susc.2011.10.025 (2012).

24 Zhang, P. *et al.* An Electrochemical in Situ Surface-Enhanced Raman Spectroscopic Study of Carbon Monoxide Chemisorption at a Gold Core−Platinum Shell Nanoparticle Electrode with a Flow Cell. *J. Phys. Chem. C* **113**, 17518-17526, doi:https://doi.org/10.1021/jp906697b (2009).
